# Beta Type Stirling Engine. Schmidt and Finite Physical Dimensions Thermodynamics Methods Faced to Experiments

**DOI:** 10.3390/e22111278

**Published:** 2020-11-11

**Authors:** Cătălina Dobre, Lavinia Grosu, Monica Costea, Mihaela Constantin

**Affiliations:** 1 Department of Engineering Thermodynamics, Engines, Thermal and Refrigeration Equipments, University Politehnica of Bucharest, Splaiul Independenței 313, 060042 Bucharest, Romania; catalina.dobre@upb.ro (C.D.); monica.costea@upb.ro (M.C.); 2 Laboratory of Energy, Mechanics and Electromagnetic, Paris West Nanterre La Défense University, 50, Rue de Sèvres, 92410 Ville d’Avray, France; mgrosu@parisnanterre.fr

**Keywords:** imperfect regeneration, numerical model, Stirling engine, thermodynamic analysis

## Abstract

The paper presents experimental tests and theoretical studies of a Stirling engine cycle applied to a *β*-type machine. The finite physical dimension thermodynamics (FPDT) method and 0D modeling by the imperfectly regenerated Schmidt model are used to develop analytical models for the Stirling engine cycle. The purpose of this study is to show that two simple models that take into account only the irreversibility due to temperature difference in the heat exchangers and imperfect regeneration are able to indicate engine behavior. The share of energy loss for each is determined using these two models as well as the experimental results of a particular engine. The energies exchanged by the working gas are expressed according to the practical parameters, which are necessary for the engineer during the entire project, namely the maximum pressure, the maximum volume, the compression ratio, the temperature of the heat sources, etc. The numerical model allows for evaluation of the energy processes according to the angle of the crankshaft (kinematic–thermodynamic coupling). The theoretical results are compared with the experimental research. The effect of the engine rotation speed on the power and efficiency of the actual operating machine is highlighted. The two methods show a similar variation in performance, although heat loss due to imperfect regeneration is evaluated differently.

## 1. Introduction

In the current energy economy context, many studies focus on renewable energy use and on the evaluation of thermal losses. Therefore, the Stirling engine draws the attention of the researchers for its many advantages, namely: strong potential of energy conversion, environmental-friendliness, quietness, and great adaptability to any type of heat source.

Study of the Stirling engine presents great complexity because of the oscillatory character of the working fluid evolutions [1]. Various thermodynamic models of Stirling engine operations have already been presented in the literature, with various assumptions. The authors of [2] aimed to develop a numerical model for a beta Stirling engine with a rhombic drive mechanism. Considering the non-isothermal effects, the thermal resistance of the heating head, and the efficiency of the regenerative channel, the energy equations can be derived for the control volumes in the expansion chamber, the regenerative channel, and the compression chamber, and can then be solved. The machine presented in the literature [3] is overhauled in the presence of heat loss and internal irreversibility, but also shows irreversibility through heat transfer. The work of [4] can be considered a key paper for the literature on Stirling engines, as well as for the identification of design methodologies for a non-proprietary Stirling engine.

The literature [5] comes with a practical validation of the practical computer simulation model of the combined plant proposed with the Otto and Stirling cycle by using finite time thermodynamics and the finite dimensional optimization methodology of thermodynamics. A model that includes frictional and mechanical losses is presented in the literature [6]—heat transfer inside the engine and other features such as auxiliary power consumption, and applies to both off-design and on-design operations. A technique for calculating the power and efficiency of Stirling machines is presented in [7]. To analyze this irreversible finite speed cycle, the direct integration of the equations based on the first law for finite speed processes is used to determine the direct power and efficiency of the cycle.

There are three modeling levels of Stirling engines [1]. The ideal analysis (or first order analysis) predicts the ideal theoretical performances of an engine [7] with a nil or unlimited convective heat transfer coefficient [8]. The uncoupled analysis (or second order analysis) takes the results of the ideal analysis and corrects them, considering a certain number of finite coefficients and losses in the engine. The obtained results are much more realistic compared with those obtained based on the first order analysis. Some relevant research done by Cullen et al. [9], Bonnet S. et al. [10], Finkelstein [11], and Walker et al. [12] adopted this approach.

The coupled analysis (or third order analysis) is based on a fine division of the engine in various control volumes, considering all of the main losses. Numerical methods have been applied to solve the equation system governing the process. It is worth noting that this model is defective because it does not properly simulate the fluid flow, particularly the turbulence, and it uses the empirical correlations to calculate the heat transfer and friction coefficients, which seem to be inadequate for obtaining accurate results. Some of the analyses at this level were developed by Chow [13], Urielli [14], Tew et al., [15], Organ [16], and Gedeon [17].

In the following sections, a finite physical dimensions thermodynamic method (FPDT; first order) and a 0D modeling (isothermal analysis) using the Schmidt model (second order) are presented.

The FPDT model [18] is based on the irreversible thermodynamics approach, which is an old approach, but it has had some improvements and engineer adjustments, which were the aim of recent papers developed by Grosu et al. [19,20,21].

The Schmidt model is an isothermal analysis that considers the same irreversibilities as the FPDT model, and in addition to this, the kinematics of the pistons are considered. This model is effectively an old one (1871), but it is chosen as a starting point by many researchers for their models because of its simplicity and its reduced time of calculation, which represent an interesting advantage in comparison with the coupled analysis.

The influence of fluid viscosity, leakage, or other types of source of losses is implicitly considered in the energetic balance scheme, even if it is not detailed in this paper. Empirical correlations could be used in the same way as C.H. Cheng and Y.J. Yu [2] and Y. Timoumi et al. [22].

This study relates to a beta type Stirling engine, comparing the experimental measurements with the analytical results of the two thermodynamic models.

## 2. *β* Type Stirling Engine

A *β*-type Stirling engine characterized by the arrangement of the piston engine, displacer, and heat exchangers in a single cylinder of highly resilient glass was analyzed. The two pistons undergo a reciprocating alternative movement with an angle of 110°. It can operate as an engine by using an electrical resistance located at the top of the cylinder, which constitutes the heat source. The cylinder is surrounded by a water jacket within which a water flow constitutes the cold sink. The displacer forces the passage of the gas from the bottom part to the top part of the cylinder, and vice versa. It also holds a highly conductive material that is used for the heat storage/release, thus playing the regenerator role.

**Figure 1:**
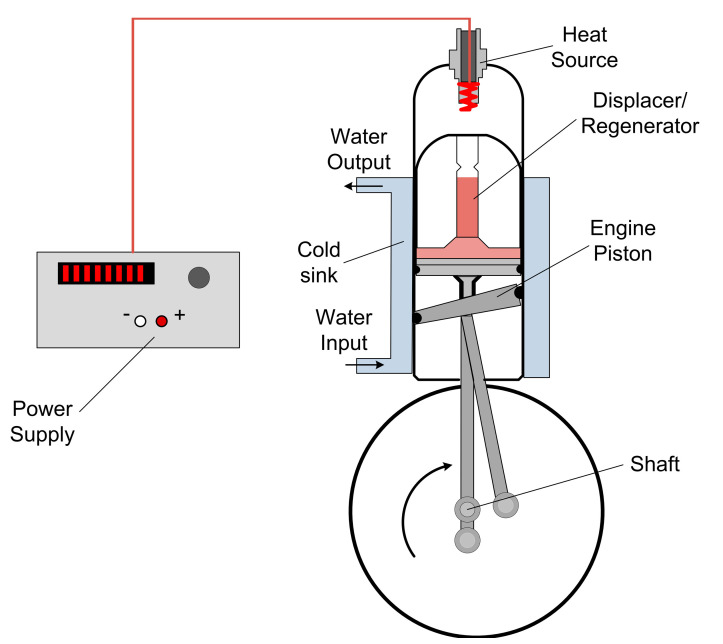
Scheme of the cylinder of the β-type Stirling engine.

The studied hot air engine configuration is presented in Figure 1.

The Stirling engine is equipped with several sensors, namely: pressure sensor, piston instantaneous position sensor, thermocouples, ammeter, voltmeter, and a device composed of photodiodes and a drilled disc, which allows for flywheel revolution speed measurement.

The working fluid is air, which is assumed to behave as a perfect gas. Any change in the fluid is not appropriate, as this system has an academic use and benefit. It can operate like an engine and provide mechanical work, or like a refrigerating machine (inverse cycle), so disassembly may be simple. The pressure charge is 1 bar and should remain 1 bar, thus this engine provides little mechanical power. The rotational speed of the engine, n, is assumed to be constant.

The heat input to the engine is provided by the electrical resistance, so heat flow rate is obtained by ${\dot{Q}}_{h}=UI$. The mechanical power is calculated by the integral of the real cycle is obtained using the instantaneous pressure and position of the working piston.

## 3. Stirling Engine Modeling

### 3.1. Finite Physical Dimensions Thermodynamics (FPDT) Method Applied to Exo-Irreversible Stirling Cycles with Imperfect Regeneration

The finite physical dimensions thermodynamics method (FPDT) [3] is a method that takes into account the finite time, finite speed and finite geometric dimensions. This method introduces the internal and external irreversibilities associated with the real processes carried out by the working gas. It is generally represented by “the analysis of the endo- and exo-irreversible cycle”.

The FDPT method takes into account the temperature pinch in the heat exchangers (finite contact time between the working fluid and the source or sink), the finite surfaces for the heat transfer (or finite conductance’s), the finite speed of movement for the mobile elements of the machine which results in a finite speed of the thermodynamic processes and the imperfect regeneration in the regenerator [23].

Martaj et al., in [21], show that machines operating on Carnot-like cycles must be described using the physical parameters of maximum pressure (*p*_max_) and maximum volume (*V*_max_), rather than the mass of the gas in the cycle, as practical problems are mainly constrained by technical and physical considerations [24], such as material mechanical resistance, material thermal resistance, bulk volume, and heat exchanger conductance and efficiency. In addition, it is essential to use the speed of revolution as the main variable, as heat and mass transfer are dependent on it in a direct manner.

The irreversibilities considered in the model presented below are those due to the temperature difference in the heat exchangers as well as to the imperfect regeneration.

**Figure 2:**
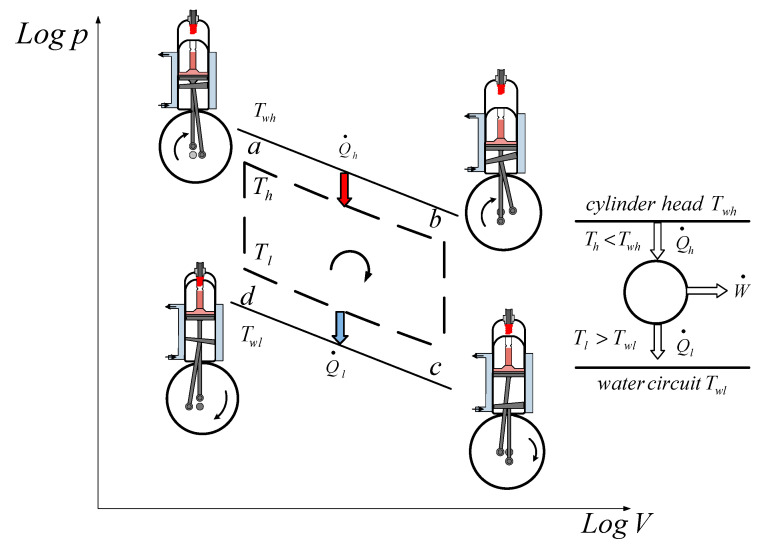
Stirling cycle engine.

The wall temperatures of the two reservoirs are *T_wh_* and *T_wl_*. The heat received by the working gas at a high temperature, *T_h_*, is *Q*_*h*.*rev*_, while that rejected at a low temperature, *T_l_*, is *Q*_*l*.*rev*_. The difference between them represents the delivered work, W, of the cycle (Figure 2).

In the case of the ideal Stirling cycle, the quantities of heat transferred in the isothermal processes in the heat exchangers are as follows:

(1) $$Q_{h.rev}=Q_{a-b}=p_{\max}V_{\max}\frac{\ln\varepsilon }{\varepsilon }=E_{\varepsilon }$$

(2) $$\left|Q_{l.rev}\right|=\left|Q_{c-d}\right|=mRT_{l}\ln\varepsilon =p_{\max}V_{\max}\frac{\ln\varepsilon }{\varepsilon }\frac{T_{l}}{T_{h}}=E_{\varepsilon }\frac{T_{l}}{T_{h}}$$

where *R* is the constant of the working gas; *m* is the mass of the working gas, which is completely transferred from the hot volume to the cold one, and in the reverse direction (the dead volume is neglected); *ε* is the compression ratio; *p*_max_ and *V*_max_ are the maximum pressure and maximum volume of the cycle; and $E_{\varepsilon }$ is the reference energy of the FPDT model.

If the regeneration is perfect, the heat stored on the regenerator during the d-a transformation and released during the reversible process b-c is as follows:

(3) $$Q_{reg\text{\_}T}=mc_{v}\left(T_{h}-T_{l}\right)=\frac{mRT_{h}}{\gamma -1}\left(1-\frac{T_{l}}{T_{h}}\right)=\frac{p_{\max}V_{\max}}{\varepsilon \left(\gamma -1\right)}\left(1-\frac{T_{l}}{T_{h}}\right)$$

where $c_{v}$ is the specific heat at a constant volume and *γ* is the adiabatic exponent of the working gas.

The imperfect regeneration $\eta_{reg}<1$ requires added heat $Q_{p,reg}$ from the source to the working gas. The same amount of heat is assumed to be rejected to the sink.

(4) $$Q_{p,reg}=E_{\varepsilon }k\left(1-\frac{T_{l}}{T_{h}}\right)=\left(1-\eta_{reg}\right)Q_{reg\text{\_}T}$$

Notation *k* is used to define the losses factor in the regenerator, as follows:

(5) $$k=\frac{1-\eta_{reg}}{\ln\varepsilon \left(\gamma -1\right)}$$

and:

(6) $$\eta_{reg}=\frac{Q_{reg\text{\_}T}-Q_{p,reg}}{Q_{reg\text{\_}T}}$$

Hence, the total heat, $Q_{h}$, delivered to the working gas is the sum of the isothermally delivered heat, $Q_{h.rev}$, and the added heat, $Q_{p.reg}$, as a result of the imperfect regeneration. The total heat, $Q_{l}$, released from the gas is the sum of the isothermally released heat, $Q_{l.rev}$, and the added heat, $Q_{p.reg}$.

(7) $$Q_{h}=Q_{h.rev}+Q_{p.reg}=E_{\varepsilon }\left[1+k\left(1-\frac{T_{l}}{T_{h}}\right)\right]$$

(8) $$\left|Q_{l}\right|=\left|Q_{l.rev}\right|+Q_{p.reg}=E_{\varepsilon }\left[\frac{T_{l}}{T_{h}}+k\left(1-\frac{T_{l}}{T_{h}}\right)\right]$$

The heat flow transferred on the hot sink and cold source can also be obtained when taking into account the engine rotational speed, *n*, as follows:

(7a) $${\dot{Q}}_{out}=n\left|Q_{out}\right|=nE_{\varepsilon }\left[1+k\left(1-\frac{T_{l}}{T_{h}}\right)\right]=K_{h}(T_{wh}-T_{h})$$

(8a) $${\dot{Q}}_{in}=nQ_{in}=nE_{\varepsilon }\left[\frac{T_{l}}{T_{h}}+k\left(1-\frac{T_{l}}{T_{h}}\right)\right]=K_{l}(T_{l}-T_{wl})$$

where *K_l_* and *K_h_* are the individual cold sources of the hot sink conductance.

The work per cycle, *W*, is the algebraic sum of the delivered heat (+) and released heat (−), or:

(9) $$\left|W\right|=Q_{h}-\left|Q_{l}\right|$$

The cycle efficiency, *η*, is given by the following:

(10) $$\eta =\frac{\left|W\right|}{Q_{h}}=\frac{\left(1-\frac{T_{l}}{T_{h}}\right)}{1+k\left(1-\frac{T_{l}}{T_{h}}\right)}$$

### 3.2. Isothermal Analysis (Schmidt Model)

The second order methods take into account the kinematics of the pistons and the internal and external irreversibilities of the machine. In addition, the non-uniformity distribution regarding the space and time [25] of the working fluid in the engine it is taken into consideration by dividing it into three, five, or more volumes [26], with which a characteristic temperature is associated. These methods use the hypothesis of independency of the energy losses.

The isothermal analysis considers the expansion and the heat exchange with the source in the same hot, isothermal volume, and the compression and the heat exchange with the sink in the same cold, isothermal volume.

**Figure 3:**
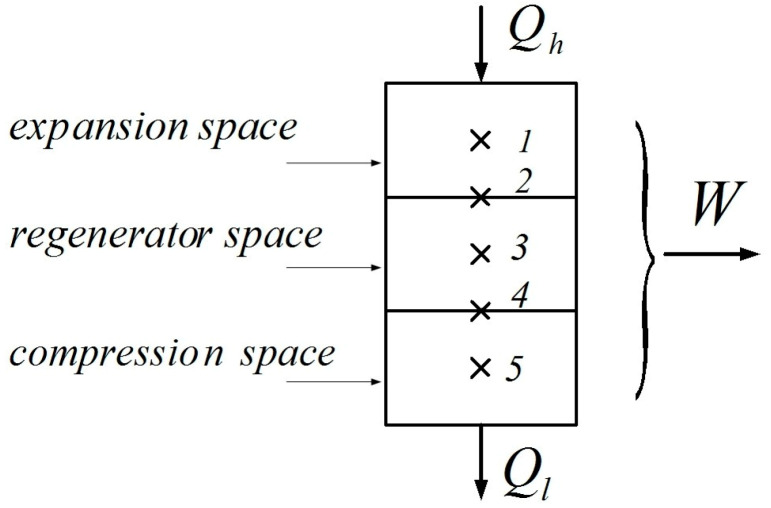
Representation of the machine spaces with their boundaries.

A classical way to model this engine with some realism is to use the Schmidt model. This analysis relies on the division of the engine into three spaces: expansion, compression, and regeneration spaces (Figure 3).

The assumptions are as follows:

- same instantaneous pressure throughout the engine,
- use of an ideal gas as a working fluid,
- constant working fluid mass,
- no leakage,
- constant cylinder wall temperature,
- harmonic/sinusoidal movement of the pistons (idealized crankshaft),
- constant temperature of the gas in the hot and cold volumes,
- constant speed of revolution,
- imperfect regeneration.

The imperfect regeneration leads to assuming that the gas temperature history will remain the same and that the part of the regeneration heat lost will be continuously compensated by a supplement of heat, $Q_{p.reg}$, provided by the source for each cycle.

The volumes of compression and expansion spaces can be expressed according to the instantaneous pistons positions by using the engine geometry [27].

The expansion (hot) space instantaneous volume has the following expression:

(11) $$V_{E}=\frac{V_{E0}}{2}\cdot \left[1-\cos(\varphi )\right]+V_{mE}$$

where *φ* is the rotation angle of the idealized crankshaft and *V_E_*_0_ is the swept expansion volume; in the case of beta type engines, this is the displacer swept volume.

The compression (cold) space instantaneous volume is a combination of several volumes, and can be expressed as follows:

(12) $$V_{C}=\left\{\frac{V_{E0}}{2}\cdot \left[1+\cos(\varphi )\right]+\frac{V_{C0}}{2}\left[1-\cos\left(\varphi -\varphi_{0}\right)\right]-V_{0l}\right\}+V_{mC}$$

where $\varphi_{0}$ is the phase lag angle of the piston movements and $V_{C0}$ is the “swept compression volume”. $V_{0l}$ is the overlapping volume in the case of a beta-type engine and is due to the intrusion of the displacer piston into the working piston swept volume.

Dead volumes, $V_{mE}$ and $V_{mC}$, on the heat exchangers are also taken into account.

The temperatures of the compression and expansion spaces are determined starting from the values obtained experimentally for the heat flow rate and the heat transfer coefficients, as follows:

- Source: ${\dot{Q}}_{losses}=h_{h}A_{h}\Delta T_{h}=h_{h}A_{h}\left(T_{h}-T_{wh}\right)$,
  where ${\dot{Q}}_{losses}$ results from the energetic balance equation, as follows: ${\dot{Q}}_{losses}={\dot{Q}}_{h}-\left|{\dot{Q}}_{l}\right|-\left|{\dot{W}}_{\exp}\right|$.
- Sink: $\left|{\dot{Q}}_{l}\right|=h_{l}A_{l}\Delta T_{l}=h_{l}A_{l}\left(T_{l}-T_{wl}\right)$,
  where $h_{h}$ and $h_{l}$ are each the hot sink cold source heat transfer coefficients.
  (13) $$\mathrm{Thus},\;T_{h}=T_{1}=\frac{Q_{losses}}{h_{h}A_{h}}+T_{wh}\;\mathrm{and}\;T_{l}=T_{5}=\frac{\left|{\dot{Q}}_{l}\right|}{h_{l}A_{l}}+T_{wl}$$
  where *T_wh_* is the wall temperature on the source (the source is inside the cylinder) and *T_wl_* is the mean temperature of the cooling water, $T_{wl}=\frac{T_{water}^{inlet}+T_{water}^{outlet}}{2}$.

The quantity of heat stored/released in the material of the regenerator depends on the value of the regeneration efficiency.

(14) $$Q_{reg}=mc_{v}\left(T_{1}-T_{4}\right)=mc_{v}\left(T_{2}-T_{5}\right)=\eta_{reg}\cdot mc_{v}\left(T_{1}-T_{5}\right)$$

Consequently, the quantity of heat that needs to be provided additionally from the heat source $Q_{p,reg}$, is as follows:

(15) $$Q_{p,reg}=mc_{v}\left(T_{4}-T_{5}\right)=mc_{v}\left(T_{1}-T_{2}\right)=\left(1-\eta_{reg}\right)\cdot mc_{v}\left(T_{1}-T_{5}\right)$$

**Figure 4:**
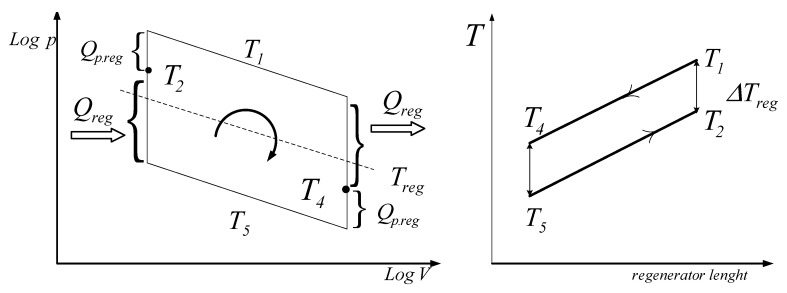
Temperature differences in the regenerator.

The internal irreversibility of this Stirling cycle is assumed to be due to the imperfect regeneration. The regenerator/displacer reciprocating movement forces the air of the cooling space towards the heating space and as well as away from it; it is also useful to store and release the heat exchanged with the regenerator material during this transfer (Figure 4). The working gas temperature at the exit of the regenerator towards the cold space $T_{4}$ is higher than $T_{5}$, and the working gas temperature at the exit of the regenerator towards the hot space $T_{2}$ is lower than $T_{1}$; the difference is the temperature gap on the regenerator $\Delta T_{reg}$, which is assumed to be constant across the whole length of the regenerator. Thus, the regenerator efficiency is defined by the following:

(16) $$\eta_{reg}=\frac{T_{1}-T_{4}}{T_{1}-T_{5}}=\frac{T_{2}-T_{5}}{T_{1}-T_{5}}=1-\frac{\Delta T_{reg}}{T_{1}-T_{5}}$$

The temperature pinch on the regenerator, $\Delta T_{reg}$, assumed to be identical at the two extreme orifices of the regenerator, can be expressed by the following:

(17) $$\Delta T_{reg}=T_{1}-T_{2}=T_{4}-T_{5}$$

In this constant-volume space, the work exchanged is null and the average temperature is supposed to be constant, $T_{reg}$.

Thus:

(18) $$\begin{array}{l} T_{4}=T_{5}+(1-\eta_{reg})\left(T_{1}-T_{5}\right)\\ T_{2}=T_{1}-\left(1-\eta_{reg}\right)\left(T_{1}-T_{5}\right) \end{array}$$

The regenerator temperature, $T_{reg}=T_{3}$, is the logarithmic average of the hot and cold space ($V_{C}$ and $V_{E}$) temperatures.

(19) $$T_{3}=T_{reg}=\frac{T_{h}-T_{l}}{\ln\frac{T_{h}}{T_{l}}}=\frac{T_{1}-T_{5}}{\ln\frac{T_{1}}{T_{5}}}$$

The fluid mass in each volume is estimated using the perfect gas law, as follows:

(20) $$\left\{ \begin{array}{l} m_{h}=\frac{V_{h}p}{RT_{1}}\\ m_{reg}=\frac{V_{reg}p}{RT_{3}}\\ m_{l}=\frac{V_{l}p}{RT_{5}} \end{array}\right.$$

and the total mass in the cylinder is as follows:

(21) $$m=m_{h}+m_{reg}+m_{l}$$

Thus, the instantaneous pressure, assumed to be uniform throughout the whole engine, is given by the following relation:

(22) $$p=\frac{1}{\frac{V_{l}}{T_{5}}+\frac{V_{reg}}{T_{3}}+\frac{V_{h}}{T_{1}}}\cdot mR$$

The elementary masses in each volume are calculated by using the following equations:

(23) $$\begin{array}{l} dm_{h}=\frac{p\cdot dV_{h}+V_{h}\cdot dp}{RT_{1}}=dm_{1},\\ dm_{reg}=\frac{V_{reg}\cdot dp}{RT_{3}}=dm_{3};\;\;\;\;dV_{reg}=0,\\ dm_{l}=\frac{p\cdot dV_{l}+V_{l}\cdot dp}{RT_{5}}=dm_{5},\\ dm_{2}=-dm_{h}=dm_{reg}+dm_{l},\\ dm_{4}=-dm_{h}-dm_{reg}=dm_{l}. \end{array}$$

The elementary masses through the interfaces and the associated temperatures are given by the following equations:

$$\begin{array}{c} dm_{2}=-dm_{h}\;\mathrm{if}\;dm_{2}>0,\;T_{2}\;=T_{1},\;\mathrm{else}\;T_{2}\;=T_{1}-\Delta T_{reg},\\ dm_{4}=dm_{l}\;\mathrm{if}\;dm_{4}>0,\;T_{4}=T_{5}+\Delta T_{reg},\;\mathrm{else}\;\;\;T_{4}=T_{5}. \end{array}$$

Taking into consideration the hypothesis of a perfect sealing, Σdm=0, so $dm_{h}+dm_{reg}+dm_{l}=0$, the pressure differential, *dp*, is given by the following equation:

(24) $$dp=\frac{-p\left(\frac{dV_{h}}{T_{1}}+\frac{dV_{l}}{T_{5}}\right)}{\left(\frac{V_{h}}{T_{1}}+\frac{V_{reg}}{T_{3}}+\frac{V_{l}}{T_{5}}\right)}$$

The elementary heat transfers in these three spaces are obtained using the energy conservation equation, as follows:

(25) $$dU=\delta W+\delta Q+h_{inlet}dm_{inlet}-h_{outlet}dm_{outlet}$$

where $h_{inlet}$ and $h_{outlet}$ are the inlet or outlet specific enthalpy on each space. Thus, elementary heats on each volume are obtained as follows:

(26) $$\begin{array}{l} \delta Q_{h}=\frac{c_{v}}{R}V_{h}dp+\frac{c_{p}}{R}pdV_{h}+c_{p}T_{2}dm_{2},\\ \delta Q_{reg}=\frac{c_{v}}{R}V_{reg}dp-c_{p}T_{2}\cdot dm_{2}+c_{p}T_{4}dm_{4},\\ \delta Q_{l}=\frac{c_{v}}{R}V_{l}dp+\frac{c_{p}}{R}pdV_{l}-c_{p}T_{4}dm_{4}. \end{array}$$

Elementary work in the compression space, $\delta W_{h}=-pdV_{h}$, and in the expansion space, $\delta W_{l}=-pdV_{l}$, allows for determining, after integration, the work provided during a cycle:

(27) $$W=W_{h}+W_{l}.$$

The cycle efficiency is given by the following:

(28) $$\eta =\frac{\left|W\right|}{Q_{h}+Q_{{}_{reg}}^{d}}$$

where $Q_{{}_{reg}}^{d}$ is an additional correcting quantity of heat to be brought by the hot source. This deficit is as a result of the masses transferred at the interface’s cold volume/regenerator and hot volume/regenerator (different densities). One notes that if $\eta_{reg}=100\%$, then $Q_{{}_{reg}}^{d}=0$.

The equations of this 0D model are solved by using Simulink tool.

## 4. Results and Discussion

The main hypothesis of this analysis is that the expansion and compression spaces are isothermal. It is also considered that the working gas is a perfect gas, and its total mass remains constant throughout the experiment (closed thermodynamic system). Using geometric and functional parameters (Table 1), measured or determined by the acquisition program (CassyLab) and using the calculation algorithm of the two studied thermodynamic methods, the following developments are obtained depending on the engine rotation speed, which is considered to be the common variable.

The initial data are listed in Table 2.

The correlation of the global heat exchange coefficient, *h*, depending on the engine rotational speed, was obtained experimentally as $h=4.0079n^{1.98}$. It has to be mentioned that this variation has the same form as the ones available in the literature [28], but is true only in the considered functioning regime. An extrapolation would not be judicious.

**Figure 5:**
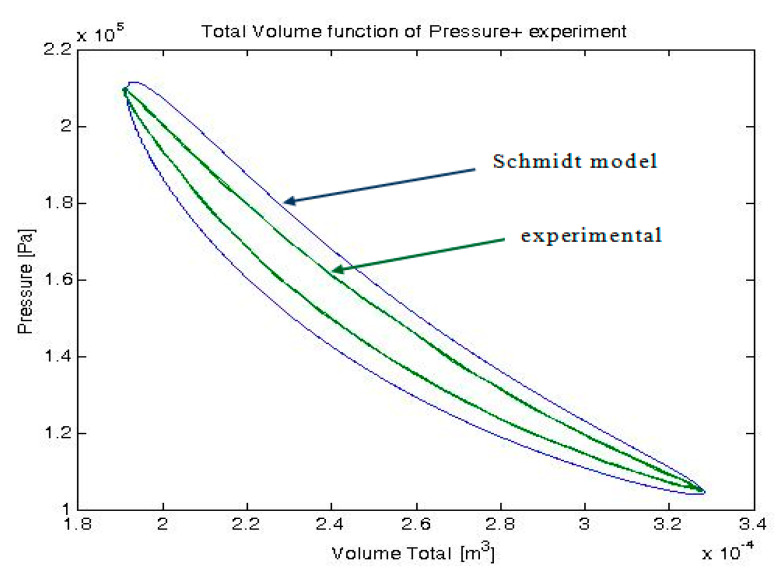
The cycle representation obtained with isothermal analysis (Schmidt model) and the experimental one in a p–V diagram.

The energies transferred and the efficiency are obtained by the two models. In addition, the instantaneous variables (temperature, pressure, volume, and mass) are given by the 0D model (Schmidt model), thus the p–V representation of the cycle could be analyzed. The indicated mechanical work was determined through the integration throughout the cycle. The p–V diagrams of the cycle obtained for the initial condition from Table 2, by isothermal analysis and by experimentation, are illustrated in Figure 5.

**Figure 6:**
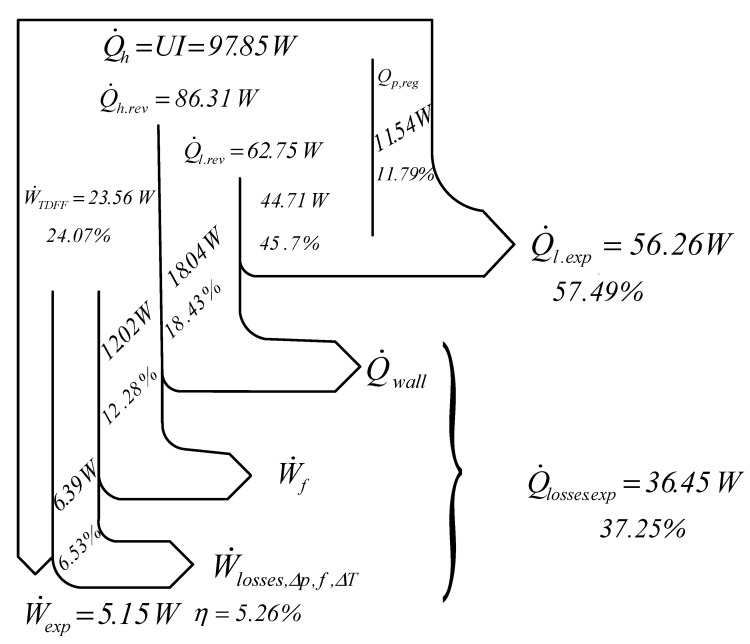
Scheme for the energetic balance (Sankey diagram).

The results obtained by applying the algorithm proposed using the FPDT method are presented in the energetic balance scheme from Figure 6.

The FPDT model and experimental results allow for determining heat flow loss and mechanical power loss due to piston–wall friction, pressure losses, friction, and temperature gap between the working gas and the heat sources. Therefore, an elaborated energy balance scheme of this particular bench test may be defined. Hence, some loss sources are identified by means of empirical correlations determined on our bench test, while others are obtained through coupling both methods (FPDT model and experiment).

where

${\dot{W}}_{f}$ is the lost mechanical power through piston–wall friction, calculated according to the relation ${\dot{W}}_{f}=0.5685n^{2.753}$, obtained on a bench test;
${\dot{Q}}_{wall}$ is the heat transfer rate lost through the cylinder wall;
${\dot{W}}_{losses,\Delta p,f,\Delta T}$ is the mechanical power loss caused by the pressure losses, friction, and temperature gap between the working gas and the heat sources, deduced from the energetic balance.

The available mechanical power, ${\dot{W}}_{FPDT}^{\prime }$, after removing the power lost through friction ${\dot{W}}_{f}$, is as follows:

(29) $$\left|{\dot{W}}_{FPDT}^{\prime }\right|=\left|{\dot{W}}_{FPDT}\right|-{\dot{W}}_{f}$$

It results in the efficiency of the following:

(30) $$\eta_{FPDT}^{\prime }=\frac{\left|{\dot{W}}_{FPDT}^{\prime }\right|}{{\dot{Q}}_{h}}.$$

**Figure 7:**
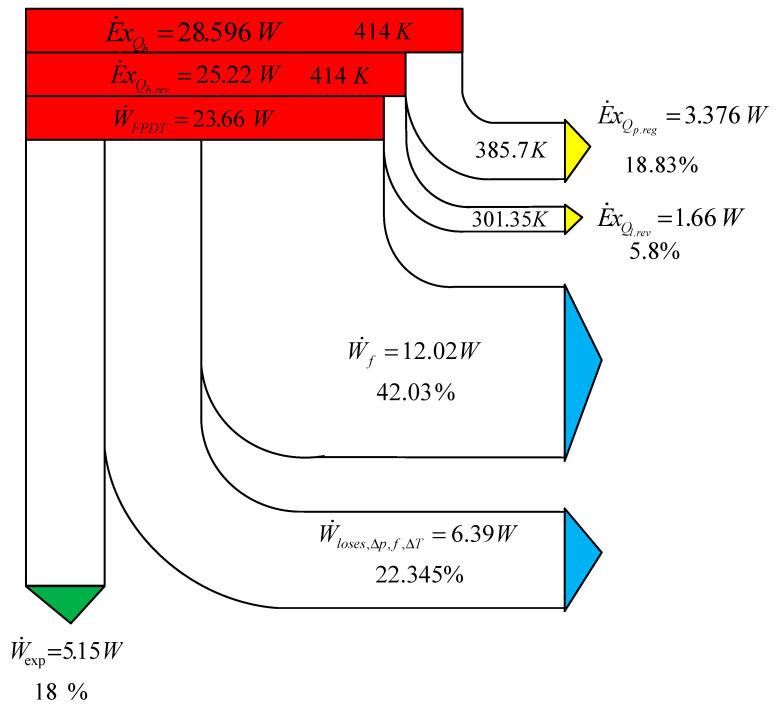
Exergy balance of the engine. Exergy distribution of the absorbed energy.

To complete the study, the exergetic analysis (Figure 7) allows for taking into account the temperature levels and estimating the different destroyed exergies due to internal and external irreversibility: imperfect heat regeneration and temperature difference between the sources and the working gas.

In the flow diagram (Figure 7), the exergy flows of the working gas with the two reservoirs (heat form hot source, *Q_h_*, and heat to cold sink, *Q_l_*) are shown at different temperatures: *T_h_* and *T_wh_* for the sources, and *T_l_* and *T_wl_* for the sink, respectively. The exergy balance of the engine shows that the regenerator imperfection loss is the biggest mechanical loss.

The comparison of the analytical study results with the experimental ones provides an overview of the machine losses. Calculation results highlight the effect of irreversibilities on the engine performance and also the influence of the engine rotational speed on them.

The results obtained by the two modeling approaches are presented in Table 3.

A Stirling engine that is not charged with high pressure can only produce a very small amount of power. For a Stirling engine to have some practical value, it has to be filled with helium or hydrogen. In this sense, a study on a changed Stirling engine would be more interesting and valuable for engineers. However, in this case, any change in the fluid is not appropriate because fact this system has academic use and benefit. It is able to operate like an engine and provide mechanical work, or like a refrigerating machine (inverse cycle), so disassembly may be simple. The pressure charge is 1 bar, and should remain 1 bar; thus, this engine provides a low mechanical power and an implicitly small efficiency.

**Figure 8:**
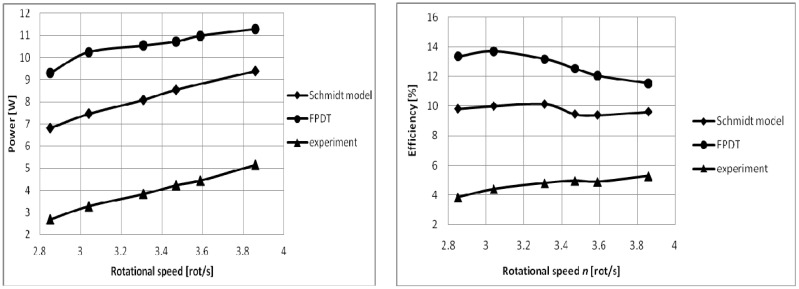
Mechanical power and efficiency versus rotational speed.

The effect of the rotational speed on the power and efficiency of the real operating machine is illustrated on Figure 8.

The isothermal analysis (Schmidt model) with imperfect heat regeneration still allows for a more judicious estimation of the mechanical power output and efficiency of the engine; this fact was revealed by comparing the analytical results with the experimental ones.

## 5. Conclusions

The work presents and discusses the dependence of β-type Stirling engine performances on the engine rotational speed using two analytical methods (finite physical dimension thermodynamics and Schmidt model with imperfect regeneration). Furthermore, the comparison of the analytical results to experimental ones provides an overview of the effect of irreversibilities on the engine performance.

The novelty introduced by the FPDT method consists of expressing the energies exchanged by the working gas as a function of practical parameters, which are necessary for the engineer throughout the design part, namely: maximum pressure, maximum volume, compression ratio, temperature of the sources, etc.

A 0D numerical model describing the variables evolution (pressure, volumes, mass, exchanged energies, and irreversibilities) as a function of the crankshaft angle is also presented. The calculated irreversibilities are due to the imperfect regeneration and temperature gap between the gas and wall on the hot and cold heat exchangers.

The results of the two thermodynamic models are presented in comparison with the experimental ones. Therefore, geometrical parameters are not considered to be variables in our models. The purpose of this study was to compare the two thermodynamic analyses and to determine which of them is closer to the experimental reality. The results are encouraging, as the two methods provided very similar results, which could be more accurate with an isothermal analysis. Note that the difference between the experimental results and the ones given by the thermodynamic models is partially justified by the fact that friction and aerodynamic losses are not taken into account in this work. Further extension of the analytical study is under development by our research team.

## Figures and Tables

**Table 1:** Dimensional data of the actual engine.

$A_{h}$	$A_{l}$	$V_{\min}\cdot 10^{-4}$	$V_{\max}\cdot 10^{-4}$	$D_{p}=D_{d}$	$S_{p}=S_{d}$	$\phi_{0}$
$\left[m^{2}\right]$	$\left[m^{2}\right]$	$\left[m^{3}\right]$	$\left[m^{3}\right]$	[m]	[m]	[°]
0.01885	0.03717	1.906	3.278	0.06	0.0484	110

**Table 2:** Initial data.

*N*	${\dot{Q}}_{h}$	${\dot{Q}}_{l}$	$T_{wh}$	$T_{wl}$	$p_{\max}$	*h*
[rot/s]	[W]	[W]	[K]	[K]	[Pa]	$\left[W/m^{2}K\right]$
3.86	97.85	56.26	414	301.35	217000	57.22

**Table 3:** Comparison of the two thermodynamic analysis results for the same initial conditions.

Experimental		FPDT		Schmidt Model	
${\dot{W}}_{\exp}$	$\eta_{\exp}$	${\dot{W}}_{FPDT}$	$\eta_{FPDT}$	${\dot{W}}_{0-D}$	$\eta_{0-D}$
[W]	[%]	[W]	[%]	[W]	[%]
5.16	5.27	11.29	11.54	9.4	9.6

## Nomenclature

*A*	heat exchange surface, m^2^
$c_{v}$	specific heat, Jkg^−1^K^−1^
*D*	diameter of the piston, m
*h*	convective heat transfer coefficient, Wm-2K-1
*I*	current, A
*k*	losses factor in regenerator, -
*K*	heat exchanger conductance, WK^−1^
*m*	mass, kg
*n*	engine rotation speed, rot.s^−1^
*p*	pressure, Pa
*Q*	heat, J
$\dot{Q}$	heat transfer rate, W
*R*	gas constant, Jkg^−1^K^−1^
*S*	stroke of the piston, m
*T*	temperature, K
*U*	voltage, V
*V*	volume, m^3^
*W*	work, J
$\dot{W}$	mechanical power, W
*Greek symbols*	
γ	adiabatic exponent, -
φ	rotation angle, °
$\varphi_{0}$	phase lag angle, °
*ε*	volumetric compression ratio (*V*_max_/*V*_min_), -
η	efficiency, -
*Subscripts*	
*C*	compression
*ε*	depending on *ε*
*E*	expansion
*d*	displacer
*h*	hot on working gas side
*l*	low on working gas side
*m*	dead
*max*	maximum
*min*	minimum
*p*	piston
*rev*	reversible
*reg*	regenerator
*v*	constant volume (specific heat)
*wl*	low on source side
*wh*	hot on sink side
